# Evolution at ‘Sutures’ and ‘Centers’: Recombination Can Aid Adaptation of Spatially Structured Populations on Rugged Fitness Landscapes

**DOI:** 10.1371/journal.pcbi.1005247

**Published:** 2016-12-14

**Authors:** Jacob D. Cooper, Benjamin Kerr

**Affiliations:** 1 Department of Biology, University of Washington, Seattle, Washington, United States of America; 2 BEACON Center for the Study of Evolution in Action, University of Washington, Seattle, Washington, United States of America; University of Texas at Austin, UNITED STATES

## Abstract

Epistatic interactions among genes can give rise to rugged fitness landscapes, in which multiple “peaks” of high-fitness allele combinations are separated by “valleys” of low-fitness genotypes. How populations traverse rugged fitness landscapes is a long-standing question in evolutionary biology. Sexual reproduction may affect how a population moves within a rugged fitness landscape. Sex may generate new high-fitness genotypes by recombination, but it may also destroy high-fitness genotypes by shuffling the genes of a fit parent with a genetically distinct mate, creating low-fitness offspring. Either of these opposing aspects of sex require genotypic diversity in the population. Spatially structured populations may harbor more diversity than well-mixed populations, potentially amplifying both positive and negative effects of sex. On the other hand, spatial structure leads to clumping in which mating is more likely to occur between like types, diminishing the effects of recombination. In this study, we use computer simulations to investigate the combined effects of recombination and spatial structure on adaptation in rugged fitness landscapes. We find that spatially restricted mating and offspring dispersal may allow multiple genotypes inhabiting suboptimal peaks to coexist, and recombination at the “sutures” between the clusters of these genotypes can create genetically novel offspring. Sometimes such an offspring genotype inhabits a new peak on the fitness landscape. In such a case, spatially restricted mating allows this fledgling subpopulation to avoid recombination with distinct genotypes, as mates are more likely to be the same genotype. Such population “centers” can allow nascent peaks to establish despite recombination. Spatial structure may therefore allow an evolving population to enjoy the creative side of sexual recombination while avoiding its destructive side.

## Author Summary

For a novel genotype to establish in a population, it must (1) be created, and (2) not be subsequently lost. Recombination is a double-edged sword in this process, potentially fostering creation, but also hastening loss as the novel genotype is being recombined with other genotypes, especially when rare. In this study, we find that spatial structure may affect both the creative and destructive aspects of recombination in rugged fitness landscapes. By slowing the spread of high-fitness genotypes, spatially restricted mating and dispersal may allow diverse subpopulations to arise. Reproduction across the borders of these subpopulations—at “sutures”—may create genetic novelty. Depending on the topography of the fitness landscape, such novelty may be in the domain of attraction of a new, higher peak; the population may “peak-jump” to an area of genotype space unlikely to be explored by mutation alone. Lineages founded by peak-jumping events are particularly prone to early extinction, as recombination with unlike genotypes may disrupt the rare allele combination and thereby produce low-fitness offspring. However, these fledgling peak lineages may be protected from early extinction by mating within small homotypic clusters—in “centers”. Thus, spatial structure may allow a population to create rare genotypes via recombination, and allow those rare genotypes to persist *despite* recombination.

## Introduction

Sexual recombination has long been a puzzling evolutionary strategy (see [1,2]). Recombination has the potential to create novel high-fitness genotypes in a population, but also to destroy high-fitness lineages by recombining them with genetically distinct lineages. Whether recombination speeds or slows adaptation depends largely on the relative strengths of its creative and destructive effects.

One of the earliest adaptive explanations for recombination is the Fisher-Muller effect, in which beneficial alleles in different lineages can recombine into a single lineage, speeding adaptation [3,4]. The Fisher-Muller effect exemplifies the creative aspect of sex, and many studies have shown faster adaptation due to Fisher-Muller dynamics [5–8]. However, the Fisher-Muller effect assumes that beneficial alleles remain beneficial when recombined into new genetic backgrounds. This assumption is necessarily broken in multi-peaked fitness landscapes [9], which arise when genetic interactions among loci yield multiple high-fitness allele combinations separated by valleys of low-fitness intermediate genotypes. In such landscapes, the adaptive effects of recombination are more complex.

Studies on two-locus rugged landscapes focus on escape from suboptimal peaks, and have found that modest levels of recombination may speed adaptation slightly, while substantial recombination slows or halts adaptation entirely [10–12]. However, studies on rugged landscapes with more than two loci yield conflicting results, variously reporting recombination as slowing adaptation [13], speeding adaptation [14], or having complex effects dependent on the topography of a fitness landscape, the population inhabiting it, and the time scale considered [15–17]. Studies on empirical fitness landscapes report recombination as speeding adaptation [6,18] or having complex effects dependent on the fitness topography and rate of recombination [15]. The varied results described above may partly depend on the genetic variation that a particular landscape supports. If there are multiple suboptimal peak genotypes, these competing lineages may interact. Depending on the topography of the fitness landscape, recombination between individuals on different suboptimal peaks may create an offspring in the attractive domain of a novel peak, termed “peak-jumping” [15,19]. Thus, in topographies that permit peak-jumping, when *sub*populations occupy different suboptimal peaks, recombination may allow peak-jumping to novel, higher peaks [19,20].

What conditions might enable a recombining population to maintain the diversity required for peak-jumping? Restricted mating and dispersal (which we call “local reproduction”) may promote population-wide diversity by slowing the spread of high-fitness genotypes and creating competitive refugia for lower-fitness genotypes [21,22]. However, the same spatial restriction that allows population-wide diversity also impedes recombination between those diverse types, as mating occurs largely within monotypic clusters. Martens and Hallatschek [22] show that recombination between spatially abutting lineages (which we call “sutures”) can be sufficient to speed adaptation due to Fisher-Muller effects in their smooth landscape model. In some rugged landscapes, recombination at sutures may allow peak-jumping. However, lineages founded by peak-jumping events are particularly prone to early extinction as recombination may disrupt the rare allele combinations and consequently prevent establishment—recombination with the majority genotype may pull fledgling peak populations off their precipices and into the valley between [23]. On the other hand, recombination within monotypic clusters (which we call “centers”) may allow high fidelity of rare allele combinations, but also prevent the creation of such rare allele combinations as no effective recombination is occurring. Which effects of sutures and centers dominate, and in what circumstances? In this paper, we examine the combined effects of recombination and local reproduction on adaptation on rugged landscapes.

## Model

In our simulation, a population inhabits an *n* × *n* square lattice. Each lattice point may be empty or may house one organism. Organisms have a haploid genotype of *L* loci, where the allele at each locus is either a 0 or a 1. Each genotype has an associated survival probability (*S*_*G*_). Unless otherwise indicated, populations are initialized with individuals of the genotype farthest from the optimal genotype (that is, *G*_0_ such that *H*(*G*_0_, *G*_*opt*_) = *L*, where *H* is the Hamming distance operator and *G*_*opt*_ is the optimal genotype), with each lattice point having an $S_{G_{0}}$ probability of starting occupied. Evolution occurs via discrete update steps described below, and simulations conclude when the optimal genotype reaches a predefined frequency, or when a predefined number of epochs have occurred, where an epoch is defined as *n* × *n* updates.

At each update, a point is chosen at random. If this focal point houses an individual of genotype *G*, the individual dies with probability 1 − *S*_*G*_, and the lattice point becomes empty. If the focal point is already empty, then a birth event can occur. For a birth event, two parents are needed. The first parent is chosen from a pre-defined dispersal neighborhood about the focal point, and second parent is chosen from a pre-defined mating neighborhood about the first parent. For simplicity, we set the sizes of these two neighborhoods equal, and call the radius of this neighborhood the “reproductive distance”. If there are no parents who satisfy the criteria, no birth event occurs. We focus on two extreme cases. In our “local reproduction” condition, a focal point’s neighborhood is defined by the lattice points immediately to the north, east, south and west (the Von Neumann neighborhood); in our “global reproduction” condition, the neighborhood is defined as the entire lattice, minus the focal point.

Once the parents are chosen, an offspring genotype is formed by recombination and mutation. To simulate recombination, one of the two parents is chosen at random to contribute the allele at the first locus, and between-locus crossover occurs with probability *r*. Thus *r* = 0 yields no crossing over, while *r* = 0.5 yields independent assortment of parental alleles. To simulate mutation, each locus of the recombined offspring’s binary genotype changes its allelic state (0→1 or 1→0) with probability *μ*. Finally, the offspring is born, and inhabits the initially-empty lattice point.

## Results and Discussion

**Fig 1:**
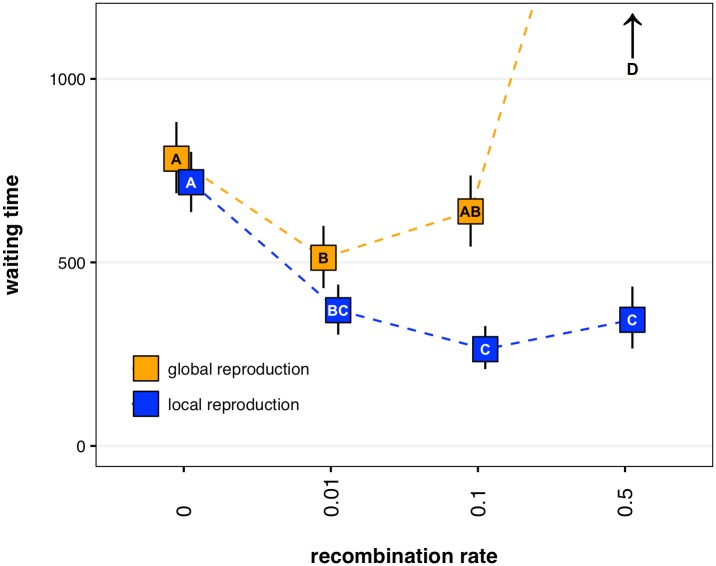
Waiting time to establishment of an optimal peak genotype at various recombination rates. We define establishment as discovery without subsequent extinction, and time as simulation epochs (see Model). Data points and error bars represent mean values and bootstrapped 95% confidence intervals of 155 replicate simulations using parameter values *n* = 70, *μ* = 0.002, *s*_*G*_ = 0.2, *s*_0000_ = 0.6, *s*_1100_ = *s*_0011_ = 0.85, *s*_1111_ = 0.9, where *G* represents all non-specified genotypes. Within each reproductive distance, data points with no shared letter are significantly different (Tukey’s HSD, *α* = 0.05). The upward arrow indicates that establishment rarely occurred by the simulation maximum of 2000 epochs.

To investigate the interplay of recombination and reproductive distance, we use a 4x2 factorial design: four recombination probabilities and two neighborhood sizes. For each factorial combination, we simulate replicate populations evolving on a multi-peaked rugged landscape. Our default fitness landscape is defined to allow peak-jumping; that is, there exist two suboptimal peaks (0011 and 1100) which can recombine to produce the optimal genotype (1111). We will relax this contrivance later in our results. In this 4x2 experiment, all populations are initialized on a suboptimal peak (0000), and all parameters (lattice size, initial density, mutation rate, *etc*.) are held constant. We find that the qualitative effect of recombination–whether it speeds or slows the traversal of the rugged fitness landscape–can depend on whether reproduction is localized (Fig 1), and this interaction between recombination and reproductive neighborhood is significant (p<0.001, Manly’s permutation test [24]). When reproduction is global, slight recombination speeds peak establishment while substantial recombination slows peak establishment. However, when reproduction is local, all rates of recombination speeds peak establishment.

To investigate why the effect of recombination may depend on reproductive distance, we focus on two aspects of a genotype's spread through a population: discovery (*i*.*e*., first appearance of the genotype in the population) and establishment (*i*.*e*., first appearance without subsequent loss). Both the discovery and establishment of the optimal genotype are affected by the interaction between recombination and reproductive distance, and the rate at which simulations discover and establish the optimal peak genotype appears to be biphasic within each parameter set (S1 Fig). The first phase, presumably due to discovery via recombination, shows rapid discoveries and subsequent establishments of the optimal genotype. The second phase, presumably due to discovery via mutation (indeed, when r = 0 this is the only phase), shows slower discoveries and a substantial lag between discovery and establishment. Both phases onset earlier when reproduction is global, yet most global reproduction simulations lag behind their local reproduction counterparts. This is because, when recombination is nonzero, the majority of local reproduction simulations discover and establish in the first phase, presumably by recombination; while the majority of global reproduction simulations discover and potentially establish in the second phase, presumably by mutation. This tortoise-hare pattern is also seen in mean relative fitnesses of populations over time: at shorter observation times, the global reproductive schemes are likely to be higher-fitness; at longer observation times, the local reproductive schemes are likely to be higher fitness (S2 Fig).

Local reproduction seems to allow quicker discovery and quicker subsequent establishment of the optimal peak. To investigate why, we focus on discovery and establishment separately. For a peak genotype to establish in a population, it must (1) be created, and (2) not be subsequently lost.

### Sutures: Local reproduction fosters the creation of novel genotypes via recombination

**Fig 2:**
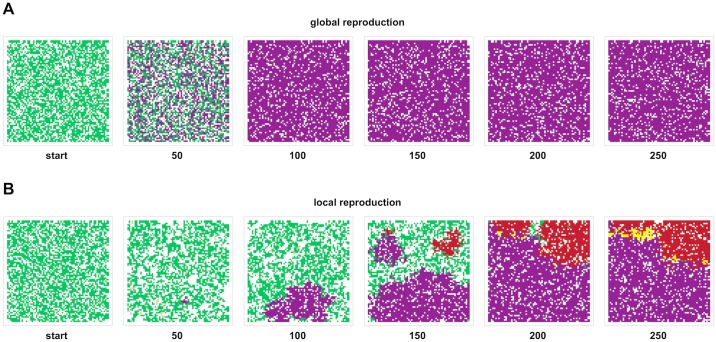
Population snapshots of representative runs from Fig 1 with a recombination rate between adjacent loci of 0.1. When reproduction is global (A), a suboptimal peak (purple) fixes by epoch 100, rendering recombination ineffective. When reproduction is local (B), two suboptimal peaks (purple and red) exist by epoch 150, and these subpopulations expand to physical proximity by epoch 200. The optimal genotype (yellow) is then created multiple times via peak-jumping at the suture between the two suboptimal peaks.

On rugged fitness landscapes, populations may become trapped on a suboptimal fitness peak. It is also possible for a population to discover multiple distinct suboptimal peaks before any single peak genotype has fixed. Localized reproduction may promote the coexistence of multiple peaks by increasing the time-to-fixation of a newly discovered peak. Thus, localized reproduction may foster the diversity of genotypes required for peak-jumping via recombination (*e*.*g*., the creation of peak genotype 1111 due to recombination between suboptimal peak genotypes 0011 and 1100) [19]. However, localized reproduction precludes peak-jumping unless the peak lineages are physically close. Physical proximity could result if two expanding peak lineages eventually abut, allowing meaningful recombination at the suture between the distinct genotypes. Such sutures between subpopulations may allow repeated discovery of genotypes in the domain of attraction of a higher fitness genotype. Indeed, in a representative simulation of intermediate recombination with local reproduction from Fig 1, multiple suboptimal peak genotypes coexist (0011 and 1100), and the globally optimal genotype (1111) is repeatedly created at the sutures between these subpopulations (Fig 2B, S1 Video). In a parallel representative run with global reproduction, no such sutures exist, because an intermediate genotype, once discovered, quickly sweeps to near fixation (Fig 2A, S1 Video).

Does local reproduction encourage sutures between subpopulations? To test this, we simulate a two-locus landscape with two peak genotypes (10 and 01) and two valley genotypes (00 and 11, the latter of which is lethal). The population is initialized on genotype 00, and we track how frequently genotype 11 is created, and how it is created. We find that genotype 11 is created by recombination more frequently in local rather than global reproductive schemes, while it is created by mutation at approximately the same frequency in the two schemes (S3 Fig).

### Centers: Local reproduction mitigates the loss of novel genotypes via recombination

Once a peak genotype is discovered, it may be lost due to subsequent recombination with unlike types, lowering the genotypic fidelity of its lineage [25]. When recombination rates are high, such loss may prevent a genotype from establishing [10,11,26]. However, spatially segregated populations may harbor population “centers”, in which mating pairs are likely to be genetically similar, preserving genotypic fidelity. Such centers may allow rare genotypes to persist in a population despite recombination. To examine the effect of centers on the establishment of a novel peak genotype, we model adaptation on a two-locus landscape in which a population may escape from suboptimal peak genotype 00 by crossing an adaptive valley (genotypes 10 and 01) to optimal peak genotype 11. We find a three-way interaction between recombination, reproductive distance, and centers (p = 0.03, Manly’s permutation test). Frequent recombination slows the establishment of the optimal peak genotype in global but not local reproductive schemes (S4 Fig, top row). However, if ‘centers’ are prohibited—that is, if a rare peak genotype (*i*.*e*., a peak genotype comprising less than 1% of the population) happens to select a homotypic neighbor as a mate, the mate is replaced with a random individual in the population—then the local and global reproductive schemes have similar results: when recombination is frequent, valley-crossing is effectively prohibited (S4 Fig, bottom row).

Nonspatial analysis of the two-locus rugged landscape suggests that valley-crossing is effectively prohibited when the recombination rate exceeds the selective advantage of the distant peak, as genetic loss due to recombination outpaces selection [10,11,26]. We too find a threshold above which valley-crossing is effectively prohibited, unless centers are provided by local recombination. The adaptive effects of local inbreeding have been investigated since at least Wright, who focused on the resultant decrease in the effective population size [27,28]. The corresponding increase in drift may allow subpopulations to cross adaptive valleys through sequential fixation [29], which may speed valley crossing for the population as a whole [30]. Here, we focus rather on the local decrease in the effective recombination rate (that is, the actual change in linkage disequilibrium due to recombination [31]) which occurs in ‘centers’, and protects rare allelic combinations regardless of their origin.

### Sutures and centers extend the parameter space in which recombination speeds adaptation

**Fig 3:**
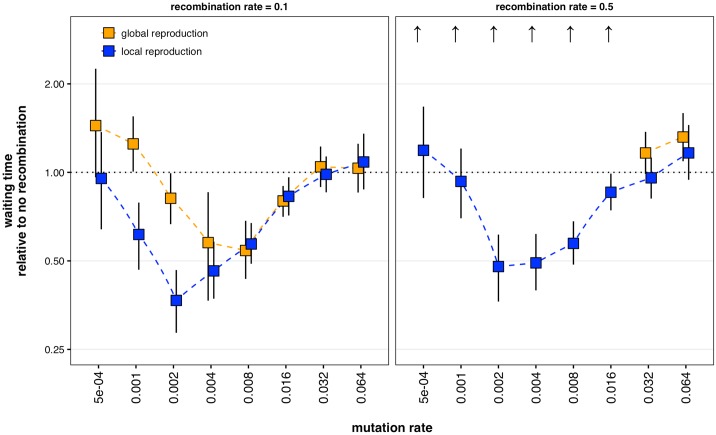
Recombination’s effect on time to optimal genotype establishment over various mutation rates. When reproduction is local, there is a larger (r = 0.1, left panel) or existent (r = 0.5, right panel) range of mutation rates in which recombination speeds establishment. Each data point represents the ratio of two means of 20–155 replicate simulations each, and error bars represent bootstrapped 95% confidence intervals of those ratios. Unless otherwise noted, all parameter values are identical to those in Fig 1. Upward arrows indicate that establishment rarely occurred in the recombining simulations by the parameter sets’ maximums of 2000 epochs for *μ* ≥ 0.002, 6000 epochs for *μ* = 0.001, and 20,000 epochs for *μ* = 0.0005.

While recombination may allow a population to more quickly climb a local peak, it can also trap populations on suboptimal peaks [17]. However, recombination may aid escape from suboptimal peaks if the landscape topography permits peak-jumping [14,19,32]. For peak-jumping to occur, multiple suboptimal peak genotypes must coexist in a population. For peak-jumping to substantially speed adaptation, distant peaks cannot be easily accessible by mutation. Thus, there is a limited range of mutation rates in which peak-jumping speeds adaptation: mutation rates must be high enough to create a diversity of genotypes, but not so high that all genotypes are easily accessible. By slowing the spread of high-fitness genotypes, local reproduction allows greater variation at lower mutation rates, and therefore expands the window in which recombination speeds adaptation (Fig 3). Similarly, larger lattices are more likely to allow variation, as more time is required for a fitter genotype to displace a less-fit genotype. Indeed, the larger the lattice, the more recombination speeds adaptation (S5 Fig).

Local reproduction promotes the coexistence of distinct types in a population, and recombination between distinct types may speed adaptation. Thus, at intermediate levels of recombination (r = 0.1), local reproduction expands the range of mutation rates for which recombination speeds adaptation. This expanded range persists at high levels of recombination (r = 0.5), while the corresponding range for global reproduction disappears entirely. Without the centers provided by local reproduction, high levels of recombination trap populations on suboptimal peaks. The protective effect of centers is robust to occasional global reproduction (S6 Fig).

Sutures should be most effective when recombination between two suboptimal peaks can create offspring in the attraction basin of a third, higher peak, allowing for peak-jumping. Centers should be most effective when novel peaks are discovered via peak-jumping, as recombination between the nascent peak and the majority genotypes can create low-fitness offspring. Thus the ability of sutures and centers to modulate the effects of recombination—to harness the creative aspect and mitigate the destructive aspect—may also be sensitive to the particular topography of a rugged landscape.

### Sutures and centers in empirically derived fitness landscapes

**Fig 4:**
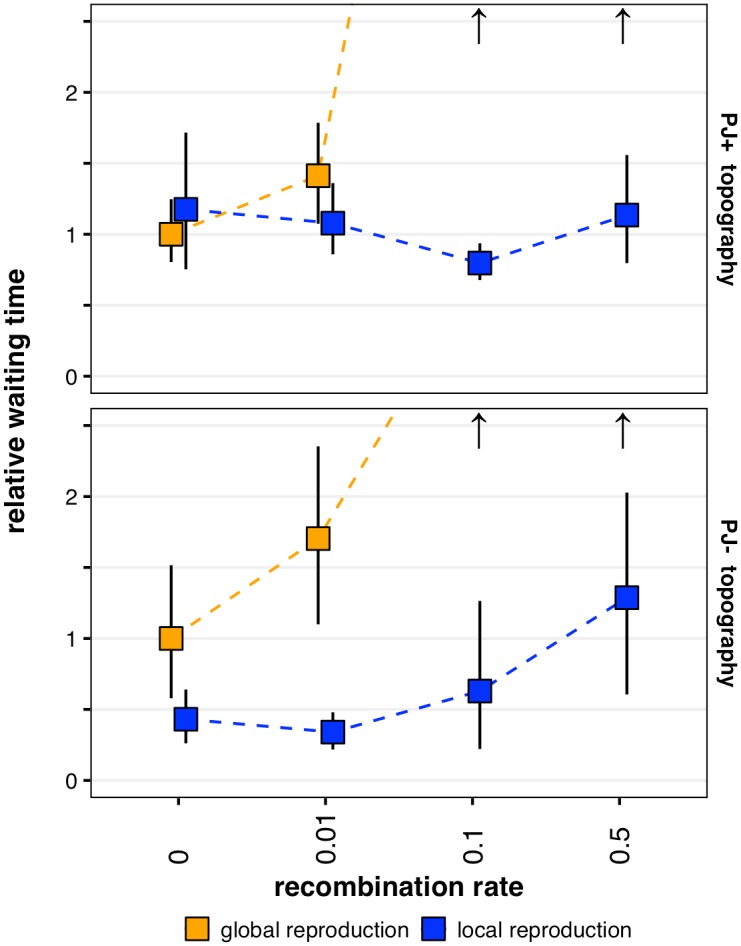
Waiting time to establishment of optimal genotypes on empirically-derived rugged landscapes at various recombination rates. When reproduction is global, recombination slows or prevents the establishment of an optimal genotype. Local reproduction mitigates the slowing effect of recombination in both landscapes. In the landscape whose topography allows recombination between suboptimal peaks to create an offspring in the attractive basin of the optimal genotype—a landscape that permits peak-jumping—recombination can speed the establishment of the optimal genotype. Data points and error bars represent mean values and bootstrapped 95% confidence limits of 15 replicate simulations using parameter values *n* = 70, *μ* = 0.001; the mutation rate was chosen to highlight the effect. Upward arrows indicate that establishment never occurred by the simulation maximum of 5000 epochs. For an explanation of the conversion from relative fitnesses (as reported in [15] to comparable survival probabilities (as used in this model), see S1 Table.

The full topographies of some naturally occurring fitness landscapes have been measured for small subsets of their genotype spaces [33]. De Visser *et al*. [15] generated 5-locus empirical fitness landscapes by introducing deleterious mutations into the asexual fungus *A*. *niger*, and measuring the fitness effects of five individual mutations and all combinations thereof. Two complete 5-locus fitness landscapes were generated, with 32 genotypes each (though the landscapes are not completely independent as they share four of their five loci of interest). Both landscapes were found to be rugged, with multiple local maxima and minima. However, only one of the landscapes (which we call PJ^+^) had suboptimal peaks which could recombine into the attraction basin of the optimal peak; the other landscape (PJ^−^) did not. De Visser *et al*. found that recombination generally slows or halts the establishment of the optimal genotype in either landscape, though there was a window of very infrequent recombination that could speed adaptation in PJ^+^ and very slightly and rarely speed adaptation in PJ^−^(see [15], supplement B1). We create landscapes parallel to PJ^+^ and PJ^−^for our model (*e*.*g*., replacing relative fitness with relative survival probabilities), and simulate evolution as before. We find a significant three-way interaction between recombination, reproductive distance, and fitness landscape topology on the waiting time for optimal genotype establishment (p<0.001, Manly’s permutation test). On PJ^+^, recombination slows or prevents the establishment of the optimal genotype when reproduction is global, but never slows or prevents adaptation when reproduction is local (Fig 4, top panel). On PJ^−^, whose topography is less conducive to landscape exploration via recombination, we find similar results to PJ^+^ when reproduction is global, but high recombination (r = 0.5) still slows the generation and establishment of the optimal genotype when reproduction is local (Fig 4, bottom panel).

In our test landscape and in two empirically-derived landscapes, sufficiently high rates of recombination prohibit the establishment of a novel high-fitness peak when reproduction is global, but this destructive side of recombination is alleviated when reproduction is local. Moreover, in landscape topographies that allow peak-jumping (our test landscape and, to a lesser extent, PJ^+^), recombination can speed the establishment of novel high-fitness peaks. Thus, the landscape topography affects the ability of local reproduction to mediate the effects of recombination: accentuating exploration via “sutures” while mitigating recombinatory destruction of rare genotypes via “centers”. We suggest the greatest effect of sutures occurs when peak-jumping is possible, and the greatest effect of centers occurs when novel peaks are created via peak-jumping. The prevalence of such topographical features and spatial restrictions—and therefore how relevant “sutures” and “centers” are to natural populations—remains an empirical question. It is possible, though, that by creating “sutures”, spatially structured populations may efficiently explore rugged landscapes via recombination, and by creating “centers”, those same populations may permit the establishment of novel peaks *despite* recombination. Spatially structured populations may therefore harness recombination’s constructive effects while mitigating its destructive effects on adaptation in rugged landscapes.

## Supporting Information

S1 Fig The proportion of 155 simulations that have discovered (dotted lines) and established (solid lines) the optimal genotype, over time. Discovery and establishment appears to be biphasic, with an early phase defined by rapid discoveries and subsequent establishments, and a late phase defined by slower discoveries and long lags before establishments. Only the second phase is seen when r = 0. Parameter values and raw data are identical to those in Fig 1.
(TIF)

S2 Fig The proportion of the 155 highest-fitness simulations (of 310 global and local reproduction simulations) that are local reproduction, over time. A value of 1.0 indicates that, at that time point, all local reproduction populations had higher mean fitnesses than all global reproduction populations. A value of 0.0 indicates the reverse. The highest fitness simulations are predominately global reproduction at early time points, but local reproduction at later time points. The long-term advantage of local reproduction increases with increasing rates of recombination. Parameter values and raw data are identical to those in Fig 1.
(TIF)

S3 Fig “Sutures” between suboptimal peaks allow landscape exploration. Populations are initialized with genotype 00 on a fitness landscape with peak genotypes 01 and 10. Lethal genotype 11 is created via recombination (green bars) frequently only when reproduction is local. Genotype 11 is created via mutation (blue bars) at a low rate at both reproductive distances. Bars represent mean values of 15 replicate simulations using parameter values *n* = 70, *μ* = 10^−5^, *f*_11_ = 0, *s*_00_ = 0.6, *s*_10_ = *s*_01_ = 0.85, *s*_11_ = 0.
(TIF)

S4 Fig Waiting time to establishment of an optimal peak genotype at various recombination rates, with and without prohibiting “centers”. Populations are initialized on suboptimal peak genotype 00, and must cross an adaptive valley to optimal peak genotype 11. Clustered genotype centers allow nascent peaks to establish despite frequent recombination. When reproduction is global, frequent recombination prevents valley-crossing. Likewise, when genotype 11 individuals are prohibited from mating with each other until they have reached a frequency of 1% (“centers prohibited” treatments), frequent recombination prevents valley-crossing. However, local reproduction with naturally occurring clusters of rare genotypes (“centers”) allows valley-crossing even with frequent recombination (top-right, shaded). Data points and error bars represent mean values and bootstrapped 95% confidence limits of 40 replicate simulations using parameter values *n* = 70, *μ* = 0.001, *s*_00_ = 0.8, *s*_10_ = *s*_01_ = 0.6, *s*_11_ = 0.9.
(TIF)

S5 Fig Recombination’s effect on time to optimal genotype establishment over various lattice sizes. Recombination speeds establishment on larger lattices, and the range of lattice sizes in which this speedup occurs is greater when reproduction is local rather than global. Each data point represents the ratio of two means of 40–155 replicate simulations each, and error bars represent bootstrapped 95% confidence intervals of those ratios. Unless otherwise noted, all parameter values are identical to those in Fig 1. Data points for global reproduction are not shown for r = 0.5 because establishment rarely occurred by the parameter sets’ maximums of 2000–15,000 epochs (larger epoch maximums correspond to smaller lattices).
(TIF)

S6 Fig The general effect of local reproduction is robust to rare instances of global reproduction. Rare global reproduction is defined as a 1/100 probability of global reproduction for each mating. Data points and error bars represent mean values and bootstrapped 95% confidence limits of 100–155 replicate simulations. All parameters and conventions are identical to those in Fig 1.
(TIF)

S1 Video Population composition through time of the simulations depicted in Fig 3. The starting genotype (0000) is represented by green; the two other suboptimal peak genotypes (0011 and 1100) are represented by red and purple, respectively; the optimal genotype (1111) is represented by yellow. All other genotypes are represented by grey.
(MP4)

S1 TableConversion of published fitness landscapes for our model.De Visser et al (2009) [15] created their empirical fitness landscapes (which they call CS1 and CS2) by measuring growth rates of all 32 relevant genotypes, and define relative fitness as a genotype’s growth rate divided by the maximum growth rate of that landscape’s genotypes. We convert these fitnesses (*ω*_*G*_) to survival probabilities (*s*_*G*_) with the formula $s_{G}=\frac{\omega_{G}}{2\overset{-}{\omega }}$, where $\overset{-}{\omega }$ is the average fitness of the landscape’s 32 genotypes.(DOCX)
