# Supplementary figures and images for: Evolution at ‘Sutures’ and ‘Centers’: Recombination Can Aid Adaptation of Spatially Structured Populations on Rugged Fitness Landscapes
Source: PLoS Comput Biol. 2016 Dec 14;12(12):e1005247. doi: 10.1371/journal.pcbi.1005247 (PMC5156365; doi:10.1371/journal.pcbi.1005247)

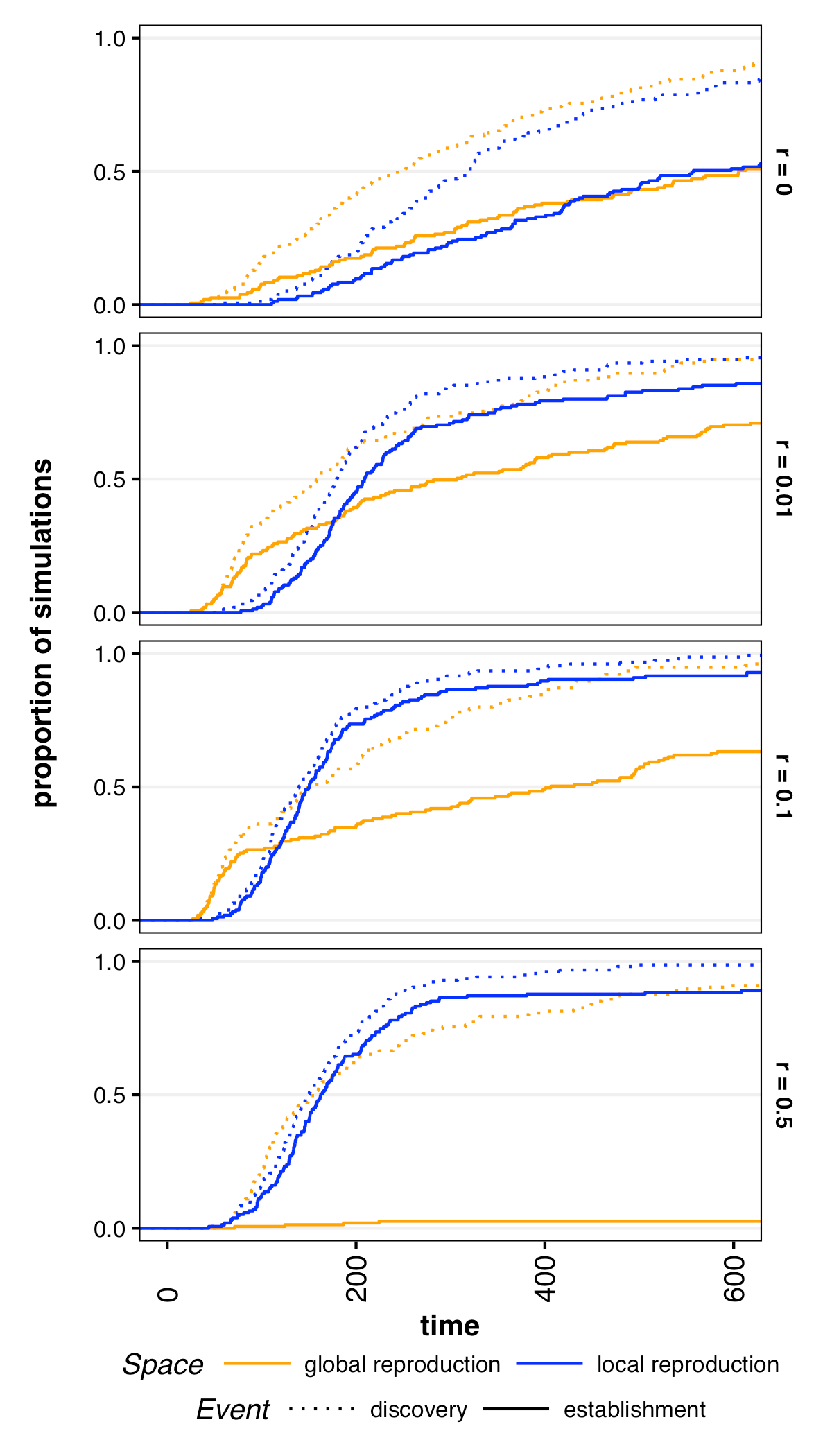

Supplement: S1 Fig — Discovery and establishment appears to be biphasic, with an early phase defined by rapid discoveries and subsequent establishments, and a late phase defined by slower discoveries and long lags before establishments. Only the second phase is seen when r = 0. Parameter values and raw data are identical to those in Fig 1. (TIF) [file pcbi.1005247.s001.tif]

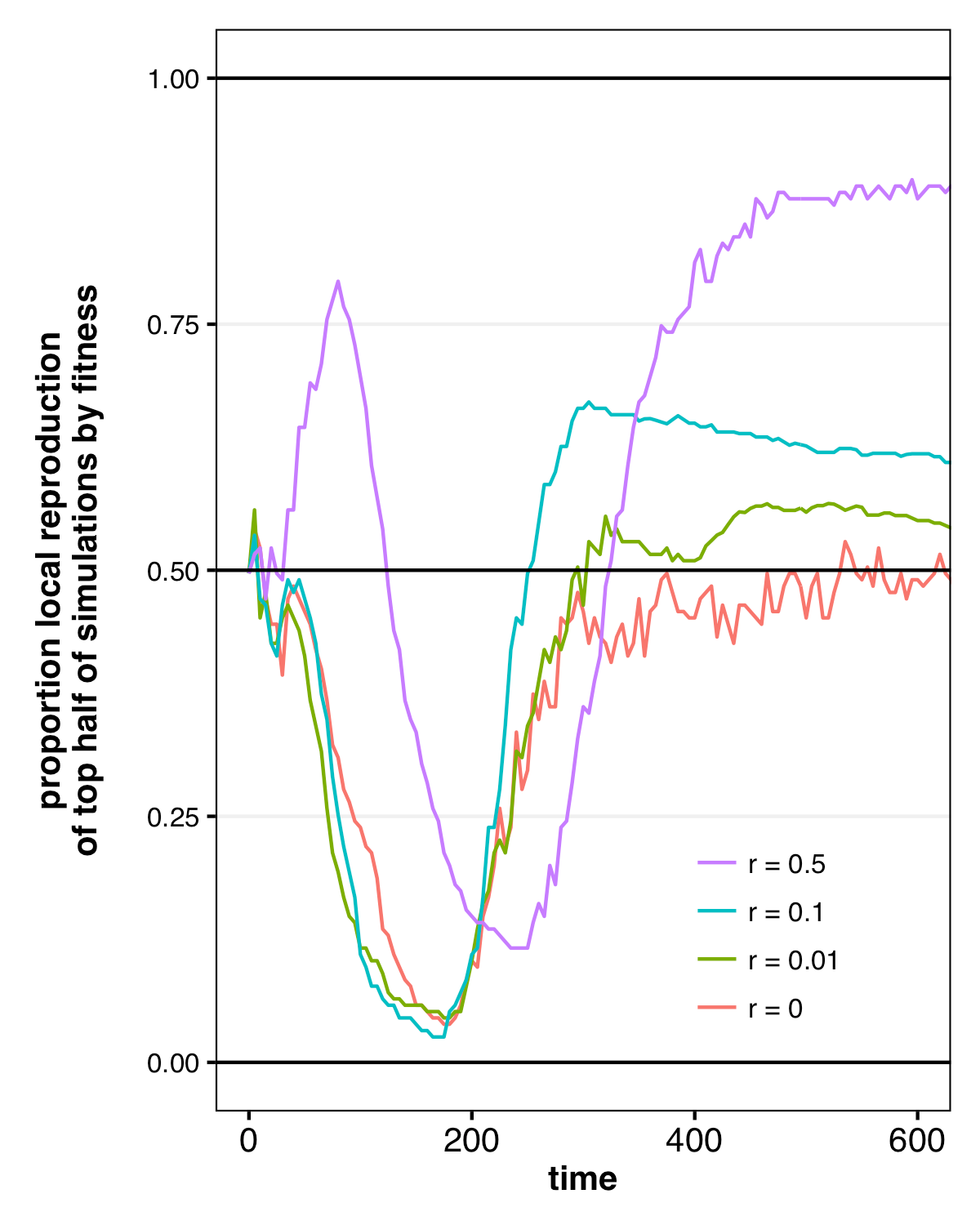

Supplement: S2 Fig — A value of 1.0 indicates that, at that time point, all local reproduction populations had higher mean fitnesses than all global reproduction populations. A value of 0.0 indicates the reverse. The highest fitness simulations are predominately global reproduction at early time points, but local reproduction at later time points. The long-term advantage of local reproduction increases with increasing rates of recombination. Parameter values and raw data are identical to those in Fig 1. (TIF) [file pcbi.1005247.s002.tif]

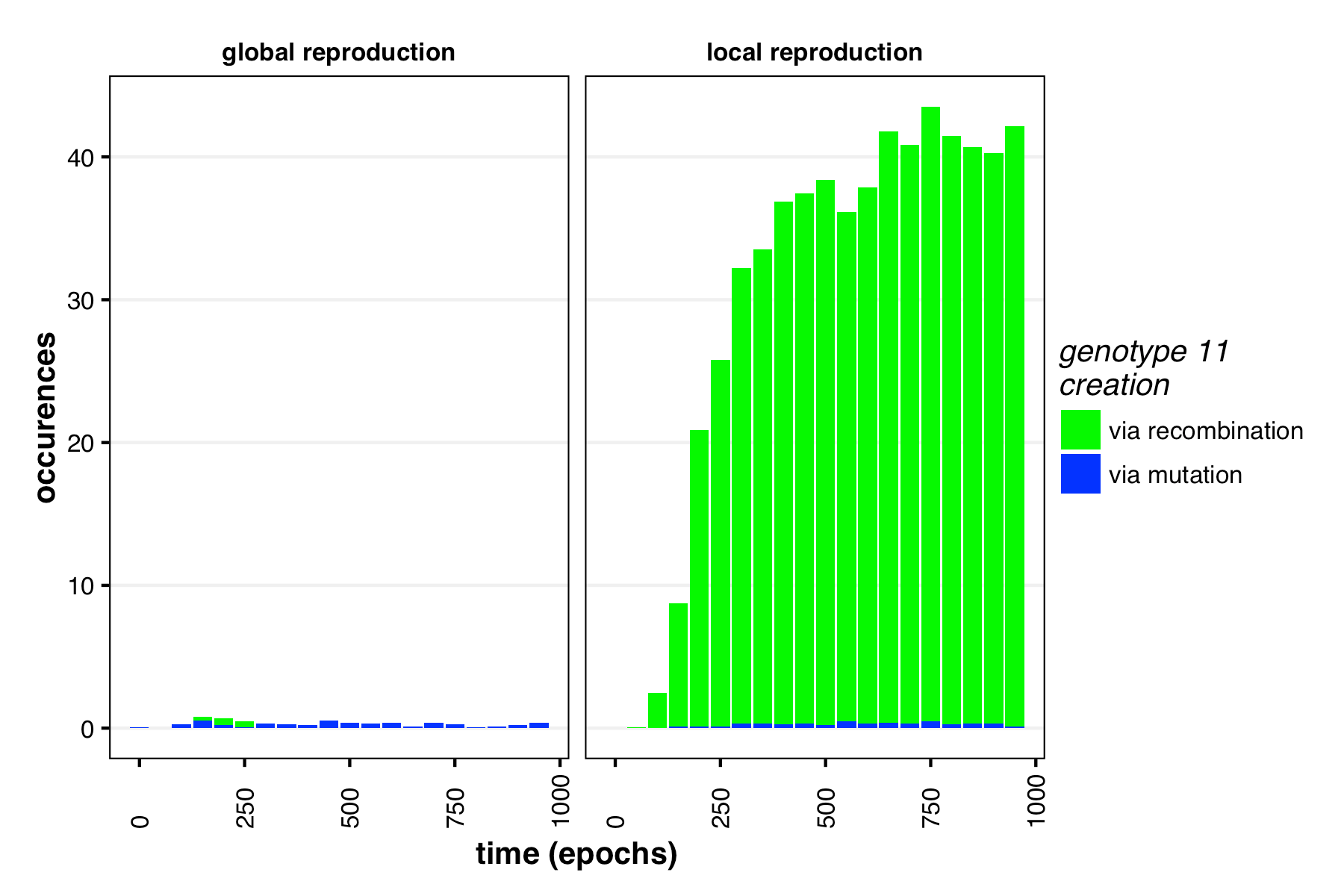

Supplement: S3 Fig — Populations are initialized with genotype 00 on a fitness landscape with peak genotypes 01 and 10. Lethal genotype 11 is created via recombination (green bars) frequently only when reproduction is local. Genotype 11 is created via mutation (blue bars) at a low rate at both reproductive distances. Bars represent mean values of 15 replicate simulations using parameter values n = 70, μ = 10−5, f11 = 0, s00 = 0.6, s10 = s01 = 0.85, s11 = 0. (TIF) [file pcbi.1005247.s003.tif]

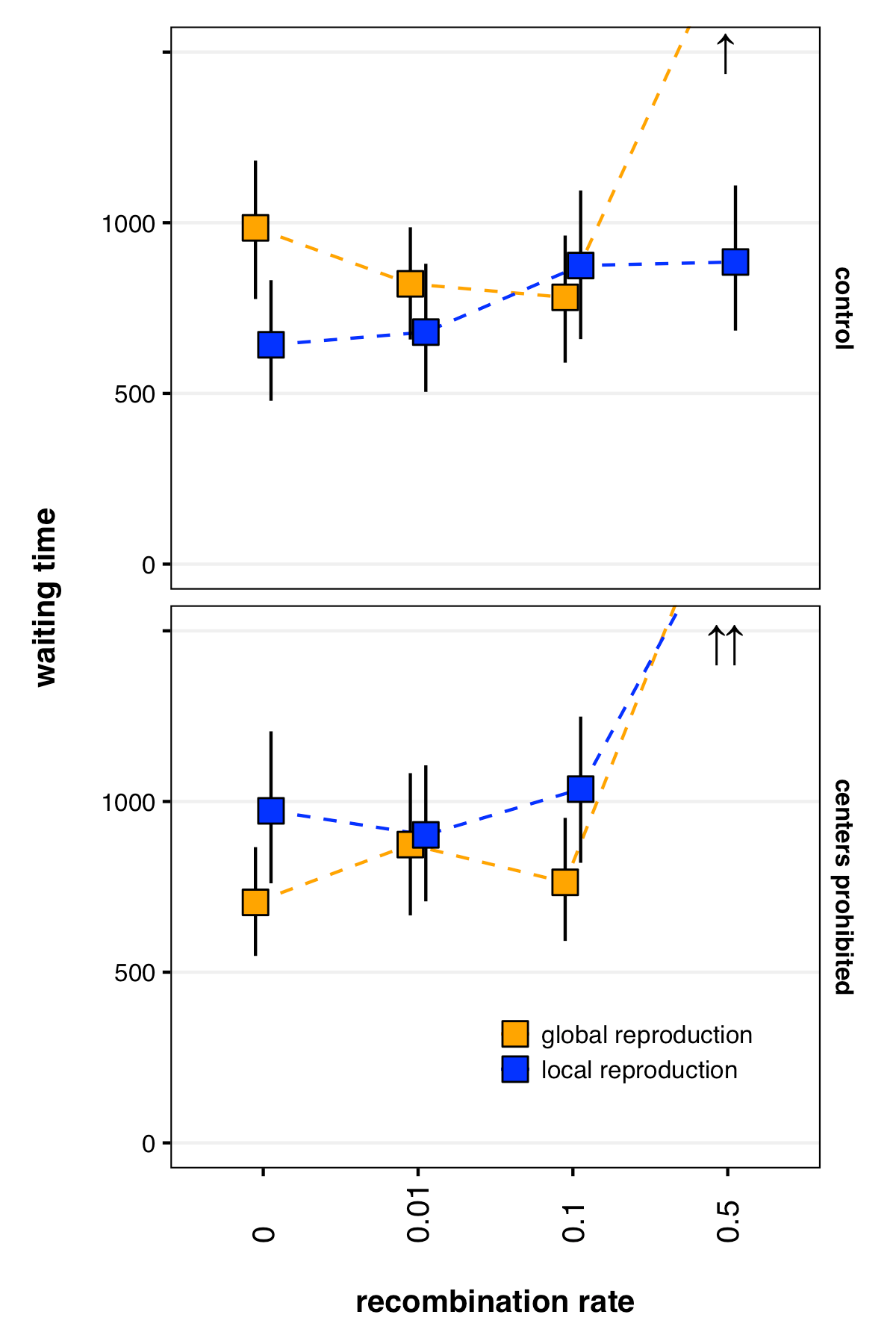

Supplement: S4 Fig — Populations are initialized on suboptimal peak genotype 00, and must cross an adaptive valley to optimal peak genotype 11. Clustered genotype centers allow nascent peaks to establish despite frequent recombination. When reproduction is global, frequent recombination prevents valley-crossing. Likewise, when genotype 11 individuals are prohibited from mating with each other until they have reached a frequency of 1% (“centers prohibited” treatments), frequent recombination prevents valley-crossing. However, local reproduction with naturally occurring clusters of rare genotypes (“centers”) allows valley-crossing even with frequent recombination (top-right, shaded). Data points and error bars represent mean values and bootstrapped 95% confidence limits of 40 replicate simulations using parameter values n = 70, μ = 0.001, s00 = 0.8, s10 = s01 = 0.6, s11 = 0.9. (TIF) [file pcbi.1005247.s004.tif]

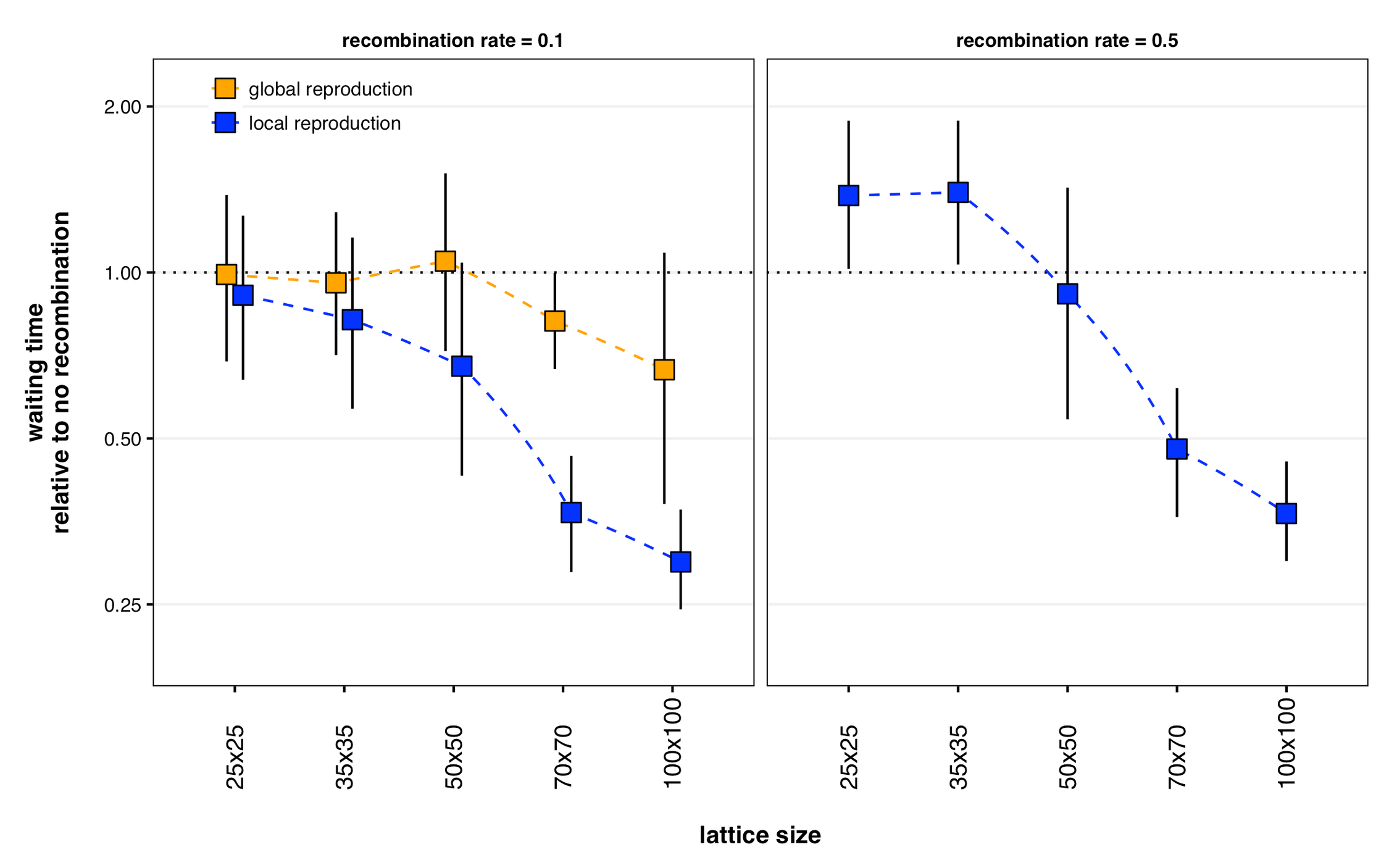

Supplement: S5 Fig — Recombination speeds establishment on larger lattices, and the range of lattice sizes in which this speedup occurs is greater when reproduction is local rather than global. Each data point represents the ratio of two means of 40–155 replicate simulations each, and error bars represent bootstrapped 95% confidence intervals of those ratios. Unless otherwise noted, all parameter values are identical to those in Fig 1. Data points for global reproduction are not shown for r = 0.5 because establishment rarely occurred by the parameter sets’ maximums of 2000–15,000 epochs (larger epoch maximums correspond to smaller lattices). (TIF) [file pcbi.1005247.s005.tif]

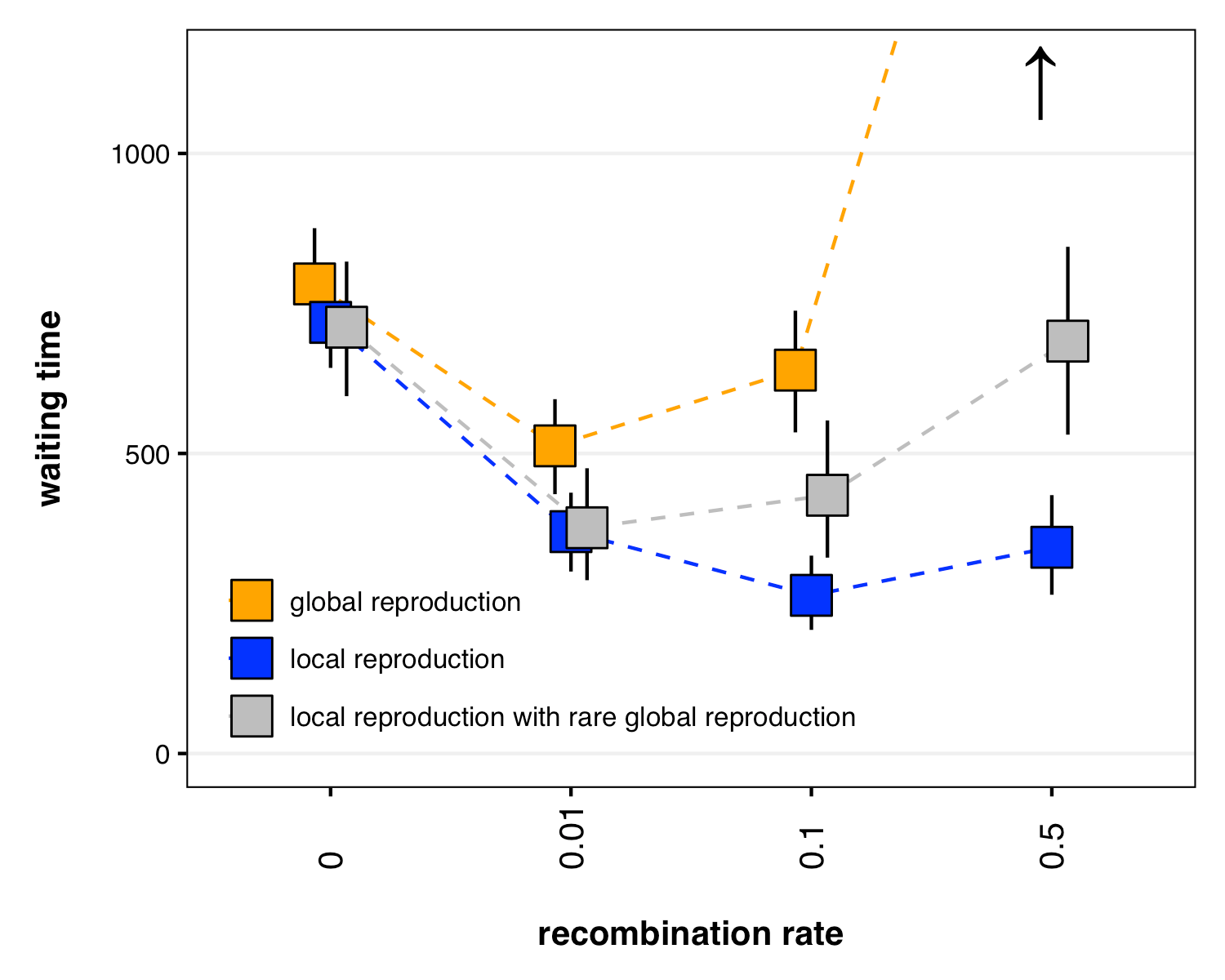

Supplement: S6 Fig — Rare global reproduction is defined as a 1/100 probability of global reproduction for each mating. Data points and error bars represent mean values and bootstrapped 95% confidence limits of 100–155 replicate simulations. All parameters and conventions are identical to those in Fig 1. (TIF) [file pcbi.1005247.s006.tif]
