# Supplementary material for: Evolution at ‘Sutures’ and ‘Centers’: Recombination Can Aid Adaptation of Spatially Structured Populations on Rugged Fitness Landscapes
Source: PLoS Comput Biol. 2016 Dec 14;12(12):e1005247. doi: 10.1371/journal.pcbi.1005247 (PMC5156365; doi:10.1371/journal.pcbi.1005247)
Supplement: S1 Table — De Visser et al (2009) [15] created their empirical fitness landscapes (which they call CS1 and CS2) by measuring growth rates of all 32 relevant genotypes, and define relative fitness as a genotype’s growth rate divided by the maximum growth rate of that landscape’s genotypes. We convert these fitnesses (ωG) to survival probabilities (sG) with the formula sG=ωG2ω-, where ω- is the average fitness of the landscape’s 32 genotypes. (DOCX) [file pcbi.1005247.s008.docx]

**S1 Table**

|  | **PJ^+^ (CS1)** | | **PJ^-^ (CS2)** | |
| --- | --- | --- | --- | --- |
| **genotype** | $\boldsymbol{\omega}_{\boldsymbol{G}}$ | $\boldsymbol{\delta}_{\boldsymbol{G}}$ | $\boldsymbol{\omega}_{\boldsymbol{G}}$ | $\boldsymbol{\delta}_{\boldsymbol{G}}$ |
| 00000 | 1.000 | 0.638 | 1.000 | 0.628 |
| 10000 | 0.878 | 0.560 | 0.878 | 0.551 |
| 01000 | 0.835 | 0.533 | 0.835 | 0.524 |
| 00100 | 0.870 | 0.555 | 0.870 | 0.546 |
| 00010 | 0.772 | 0.493 | 0.909 | 0.571 |
| 00001 | 0.793 | 0.506 | 0.772 | 0.485 |
| 11000 | 0.865 | 0.552 | 0.865 | 0.543 |
| 10100 | 0.854 | 0.545 | 0.854 | 0.536 |
| 10010 | 0.773 | 0.493 | 0.923 | 0.580 |
| 10001 | 0.873 | 0.557 | 0.773 | 0.485 |
| 01100 | 0.816 | 0.521 | 0.816 | 0.512 |
| 01010 | 0.716 | 0.457 | 0.852 | 0.535 |
| 01001 | 0.848 | 0.541 | 0.716 | 0.450 |
| 00110 | 0.778 | 0.497 | 0.855 | 0.537 |
| 00101 | 0.820 | 0.523 | 0.778 | 0.488 |
| 00011 | 0.972 | 0.620 | 0.785 | 0.493 |
| 11100 | 0.816 | 0.521 | 0.816 | 0.512 |
| 11010 | 0.748 | 0.477 | 0.879 | 0.552 |
| 11001 | 0.832 | 0.531 | 0.748 | 0.470 |
| 10110 | 0.749 | 0.478 | 0.942 | 0.592 |
| 10101 | 0.792 | 0.506 | 0.749 | 0.470 |
| 10011 | 0.753 | 0.481 | 0.795 | 0.499 |
| 01110 | 0.617 | 0.394 | 0.858 | 0.539 |
| 01101 | 0.810 | 0.517 | 0.617 | 0.387 |
| 01011 | 0.643 | 0.410 | 0.724 | 0.455 |
| 00111 | 0.671 | 0.428 | 0.745 | 0.468 |
| 11110 | 0.690 | 0.440 | 0.825 | 0.518 |
| 11101 | 0.855 | 0.546 | 0.690 | 0.433 |
| 11011 | 0.649 | 0.414 | 0.665 | 0.418 |
| 10111 | 0.692 | 0.442 | 0.686 | 0.431 |
| 01111 | 0.643 | 0.410 | 0.640 | 0.402 |
| 11111 | 0.645 | 0.412 | 0.622 | 0.391 |
| **mean** | **0.783** | **0.500** | **0.796** | **0.500** |
| **SD** | **0.095** | **0.061** | **0.095** | **0.060** |
